# Organization of Enzyme Concentration across the Metabolic Network in Cancer Cells

**DOI:** 10.1371/journal.pone.0117131

**Published:** 2015-01-26

**Authors:** Neel S. Madhukar, Marc O. Warmoes, Jason W. Locasale

**Affiliations:** 1 Tri-Institutional Program in Computational Biology and Medicine, Cornell University, Ithaca, New York, Weill Cornell Medical College, New York, New York, Memorial Sloan-Kettering Cancer Center, New York, New York, United States of America; 2 Division of Nutritional Sciences, Cornell University, Ithaca, New York, United States of America; Laurentian University, CANADA

## Abstract

Rapid advances in mass spectrometry have allowed for estimates of absolute concentrations across entire proteomes, permitting the interrogation of many important biological questions. Here, we focus on a quantitative aspect of human cancer cell metabolism that has been limited by a paucity of available data on the abundance of metabolic enzymes. We integrate data from recent measurements of absolute protein concentration to analyze the statistics of protein abundance across the human metabolic network. At a global level, we find that the enzymes in glycolysis comprise approximately half of the total amount of metabolic proteins and can constitute up to 10% of the entire proteome. We then use this analysis to investigate several outstanding problems in cancer metabolism, including the diversion of glycolytic flux for biosynthesis, the relative contribution of nitrogen assimilating pathways, and the origin of cellular redox potential. We find many consistencies with current models, identify several inconsistencies, and find generalities that extend beyond current understanding. Together our results demonstrate that a relatively simple analysis of the abundance of metabolic enzymes was able to reveal many insights into the organization of the human cancer cell metabolic network.

## Introduction

Metabolism constitutes a fundamental component of cell physiology. It allows for the processing of nutrients through chemical reaction networks, resulting in the production of energy and biosynthetic components and regulation of signal transduction processes by affecting the levels of metabolites that control the activity of proteins. Its function is essential for human health and its aberrant status is a hallmark of many diseases[1,2]. A quantitative, predictive understanding of metabolism has countless possibilities[3–5], but has been limited by the lack of available data at the level of metabolite levels, enzyme expression, and flux.

One major limitation in this systems level understanding stems from the lack of quantitative measurements of protein abundance[6]. The absolute protein concentration is essential for understanding enzyme kinetics and therefore flux through a metabolic pathway[7]. For any chemical reaction involving an enzyme, the rate of that reaction is proportional to the enzyme abundance, thus the absolute concentration of an enzyme places bounds on metabolic flux and serves as a reasonable estimate of its activity and, when compared to other enzymes in competition for a substrate, can be used as an estimate of the relative usage of that substrate. Other factors such as the Michaelis constant and turnover rates are also important but each of these parameters is independent of enzyme concentration. Thus an analysis of protein concentrations across and within pathways and, in comparison to enzymes that utilize the same substrate, can give estimates of relative fluxes emanating from the compared enzymes.

Previous studies have carried out extensive analyses of the transcriptional abundance of metabolic genes[3]. These studies have focused on changes that accompany oncogenic transformation and have uncovered insights into the pathways, genes, and reactions that are altered[1,3,8–11]. Nevertheless it has also been shown that there is, in many cases, only a modest correlation between transcript and protein abundance owing to many factors—such as translational regulation and protein stability—that influence the relationship between mRNA and protein[12–14]. Furthermore, these studies have focused on tumor-normal comparisons instead of the concentration distributions across the network, which has a different, but nonetheless relevant, biology associated with its analysis.

Advances in mass spectrometry based proteomics have allowed for in depth identification and quantitation of mammalian proteomes from biological samples[15–17]. These technologies have also allowed for estimates of protein abundance from biological samples using regression modeling. With these data at hand, it is then possible to assess the distribution of protein concentrations across the metabolic network and to make quantitative evaluations of enzyme abundance across pathways, at branch points where metabolic fluxes diverge, and for enzymes that utilize common substrates. We therefore conducted this analysis and uncovered several surprising results pertaining to the organization of protein concentrations across the human metabolic network.

## Methods

### Curation of a Metabolic, Pathway-Based, Proteome

To begin our analysis we considered the NCI-60 cell line panel which is a set of cell lines developed and maintained by the National Cancer Institute that has been used extensively for integrated molecular analyses and drug sensitivity profiling[18]. The proteomic quantification for the NCI-60 panel makes use of a standardized cell protein copy number (CPC) metric (see S1 Table), which is derived from the Label Free Quantification (LFQ) quantification from Gholami et al. [19]. The NCI-60 proteome dataset containing the LFQ data is freely available at http://wzw.tum.de/proteomics/nci60. We began by filtering out proteins in the CPC dataset that were detected in only one sample or tissue type. These sample proteins also contained a far lower average CPC than all other samples (~3000 CPC vs 71,000 CPC) indicating that they might not be relevant for making global predictions on metabolism. Each protein in this list was mapped to 86 known metabolic pathways using the Kyoto Encyclopedia of Genes and Genomes (KEGG, Release 69.0, January 1, 2014) mapping of genes to pathways. Any protein that was included in at least one pathway was included as part of the metabolic proteome. The average CPC values across all cell lines for each metabolic and non-metabolic protein were used to calculate the metabolic percentage. The pathway percentages were calculated in a similar manner, though, one protein could be involved in multiple pathways, and thus the sum of all percentages does not necessarily equal 100%. In order to calculate the various probability distribution functions of different protein classes, all of the CPC measurements across cell lines were inputted into PRISM (version 6.03), with a standardized analysis computing bin centers.

### Glycolysis Pathway Analysis

For each protein involved in the Glycolysis/Gluconeogenesis pathway from the KEGG database, the distribution was classified as the CPC values across all cell lines. The core glycolytic pathway was defined as the progressive steps through which glucose entering glycolysis is converted to lactate or can be diverted to multiple different biological pathways. In order to visualize the differences in protein counts throughout core glycolysis, a pathway was created in CytoScape (version 3.1.1) with the size of nodes corresponding to the average CPC of the respective proteins[20,21].

### Branch Point Analysis

In order to isolate metabolic pathways containing branching steps, we utilized the Recon 2.2 stoichiometry matrix to separate pathways where a single metabolite acted as a reactant to multiple different reactions—these were defined as our branches[22]. Since some branches could involve more than one reactant metabolite, we excluded any repeats our search produced. For each set of branches (each set having the same reactant metabolite) we only included ones where all catalyzing enzymes were included in our metabolic proteome. We calculated two metrics from the CPCs of these branching enzymes, each based on the number of considered enzymes. For all branching pathways we ranked the involved enzymes based on their average CPC across all cell lines, with the branching divergence (BD) score defined as:

$$\frac{CPC(Enz_{n=1})-CPC(Enz_{n=2})}{CPC(Enz_{n=1})}\text{n = rank based on CPC (descending order)}$$

$$\frac{CPC(Enz_{n=1})-CPC(Enz_{n=2})-CPC(Enz_{n=3})}{CPC(Enz_{n=1})}\text{n = rank based on CPC (descending order)}$$

For branching points where there were at least three distinct product possibilities, we redid this calculation, this time including the top three ranked enzymes instead of simply the top 2.

We then separated the branches into 2 additional categories—one way leaning pathways where branching scores produced a score above. 8, and equally distributed pathways where the top 2 BD score was less than. 2 or the top 3 BD score was less than 0. Branches were filtered to make sure no reactions were repeated in each set. For both sets of branches, either the top 2 or top 3 involved enzymes—dependent of which type of score was used to differentiate them—were separated and mapped to their respective KEGG pathways. For each set, the number of enzymes falling into each pathway was summed and a Fischer’s Exact Test was performed to see if there were any pathways that differed significantly across the two branching sets. Ratios of counts were computed by dividing the number of involved One Sided enzymes by the number of Equally Distributed Enzymes for each of the KEGG pathways.

### Cofactor Analysis

We defined oxidoreductases as proteins that either had NADP+/NADPH or NAD+/NADH as the reactants or products. Both reactants and products were included so as to allow for proteins with reversible reactions. EC Numbers of appropriate reactions were extracted using the BRaunschweig ENzyme DAtabase (BRENDA, Release 2014.2, July 2014) database and then mapped to their appropriate Uniprot (Release 2013_11) or Entrez (release date December 15^th^ 2013) identifiers [22]. We performed a similar analysis for aminotransferases, replacing NAD compounds with α-ketoglutarate in order to track the use of nitrogen throughout the cell. A graphically rich representation of cofactor usage was created using CytoScape.

### Kinetic Parameter Analysis

For each metabolic protein we used the BRENDA Database to extract all experimentally reported K_M_ values. The Simple Object Access Protocol (SOAP) interface was used to obtain kinetic properties for all metabolic proteins. We excluded all values not referenced as a wild-type experiment (such as K_M_ values obtained after a protein mutation) and further refined the list by excluding any outlier measurements. We then used a previously published list of available ΔG° values[23] plotted the log_10_(CPC), log_10_(K_M_), ΔG° values for proteins for which we obtained all three of these variables (see S2 Table). For the connectivity analysis, CPC, ΔG° and K_M_ values were scaled between 0 and 1, subsequently visualized in CytoScape and manually distributed into four groups.

## Results

### Global Analysis of Metabolic Proteome

**Fig 1:**
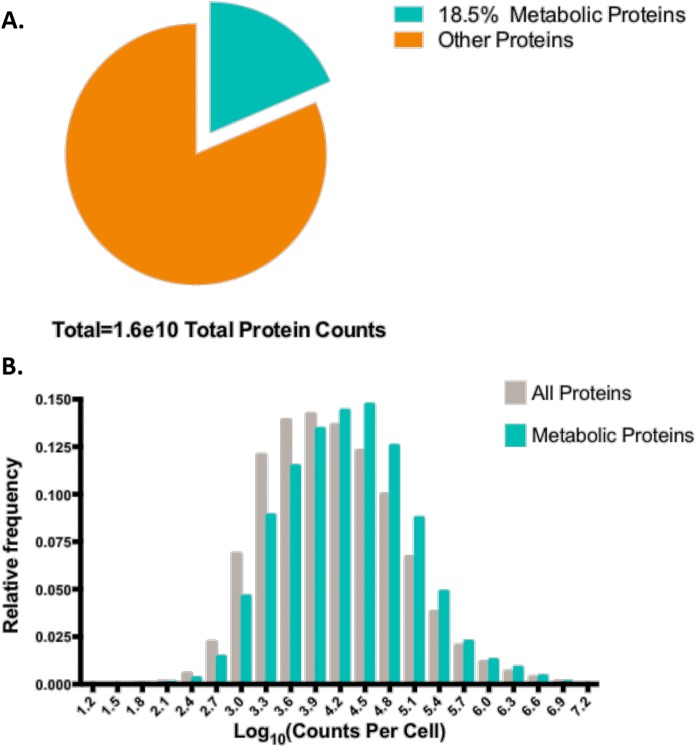
Global intracellular distribution of metabolic protein concentrations across all cell lines. **(a)** Pie chart diagraming the total percentage of metabolic proteins across all cell lines. Metabolic proteins are differentiated by a different color inset. **(b)** Probability distribution function of cell protein copy (CPC) values for all proteins and the metabolic subset—different subsets are denoted by different colors. Log_10_ values were binned with a bin difference of 10^0.3^ and the relative frequency, or percentage of values falling into that bin, were plotted.

To investigate the expression and quantification of metabolic proteins we first assembled the subset of proteins involved in metabolism. We considered a recent data set that utilized deep proteomic measurements across the NCI-60 cell line panel and a regression model to estimate protein abundance from mass spectrometry data (S1 Table)[19]. We defined metabolic proteins as any protein assigned to a known metabolic pathway in the KEGG database [3,24]. Quantifying the relative size of this subset, we found that on average, across the 59 cell lines measured, the metabolic component accounted for approximately 18.5% of the total proteome (Fig. 1a). Upon examining the distribution we also observed a roughly log normal distribution and found that the distribution of metabolic proteins followed roughly the same pattern as that of the overall protein distribution (Fig. 1b).

**Fig 2:**
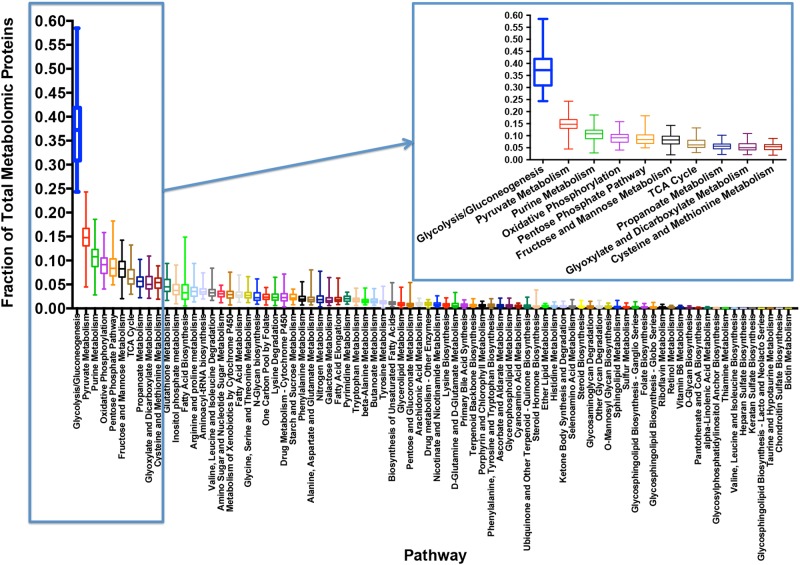
Distribution of metabolic proteome percentage across all cell lines for various pathways. For each distribution the median, standard deviations, max, and min are indicated. Inset contains a magnified look at the pathways with the highest percentages of the metabolic proteome.

Examining the proteins comprising metabolic enzymes, we sorted proteins according to the 86 metabolic KEGG metabolic pathways they were assigned to. Because a single protein is often involved in multiple biochemical reactions, we did not restrict each protein to single pathway, but counted each protein across each of the pathways to which the enzyme belonged. Of the 86 KEGG pathways, we found 6 pathways did not contain any detected proteins and did not further consider those pathways. Based on this division we were able to quantify the total protein counts and fraction of all proteins that were involved in a specific pathway across the set of cell lines (Fig. 2). Quite dramatically, the concentrations of glycolytic enzymes were found to be very large and in total resulted in nearly half of the total amount of protein partitioned into metabolic enzymes in cells. This finding likely confirms the assumption that the bulk of metabolic flux occurs within glycolysis and central carbon metabolism[1,25–29]. Importantly enzymes involved in the Citric Acid Cycle (TCA) were typically an order of magnitude or two lower in expression than those in glycolysis and these numbers place bounds on the flux that can be maintained in each of these pathways. Other pathways whose substrates are immediately derived from glycolytic carbon—such as the pentose phosphate pathway and those involving fatty acid synthesis—also had much lower protein concentrations than those of glycolysis.

### Analysis of Glycolysis

**Fig 3:**
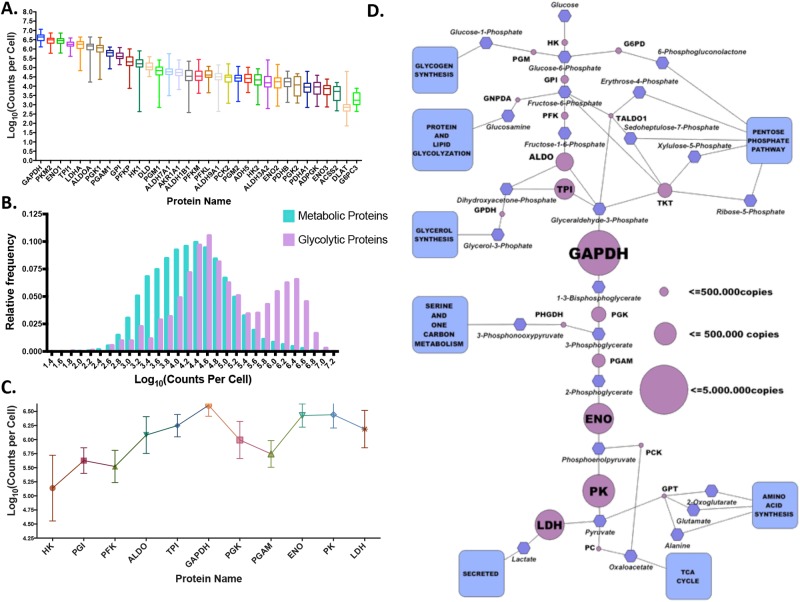
Global profile of glycolytic enzyme concentrations across all cell lines. **(a)** Distribution of cell protein copy (CPC) values for all glycolytic/gluconeogenic proteins across all cell lines. For each protein the median, standard deviations, max, and min are indicated. **(b)** Probability distribution function of CPC values for all metabolic proteins and the glycolytic/gluconeogenic subset—different subsets are denoted by different colors. Values were binned with a bin difference of 10^0.2^ and the relative frequency, or percentage of values falling into that bin, were plotted. **(c)** Distribution of 11 glycolytic proteins in a sequential pathway order. Center dot indicates average CPC value for each protein with bars indicating the max and min measurements across all cell lines. **(d)** Pathway diagram of glycolysis activity. Blue squares indicate branching into other biological pathways, blue hexagons indicate intermediate metabolites, and purple circles indicate reacting enzymes. Size of purple circle is proportional to the average CPC value for that enzyme across all cell lines.

We observed that the proportion of the proteome that cancer cells devote to glycolysis dwarfs the amount protein devoted for other metabolic pathways (Fig. 2). This is especially notable considering that the only 32 proteins were assigned to the glycolytic pathway whereas pathways such as Purine Metabolism, Pyrimidine Metabolism, and Oxidative Phosphorylation were assigned upwards of 60 proteins. Although variability exists across cell lines, this analysis suggests that glycolytic proteins can account for up to 10% of all proteins in a cancer cell. We also observe a bimodality to the distribution of all glycolytic proteins that arises from an abundance of highly expressed proteins and some isoforms exhibiting smaller expression levels (Fig. 3b). This observation then motivated us to further investigate the organization of protein expression in glycolysis. We examined enzymes along the pathway in the order that the enzymatic reactions occur. Interestingly, a non-monotonic pattern emerges in the progression of enzyme levels as glucose is metabolized through glycolysis to yield lactate, with a peak at the phosphorylation of glyceraldehyde-3-phosphate to 1,3-biphosphoglycerate—catalyzed by glyceraldehyde-3-phosphate dehydrogenase (GAPDH)—and additional later peak occurring at the conversion of 2-phosphoglycerate to phosphoenolpyruvate—catalyzed by enolase (Fig. 3c). This apparently non-random pattern likely has biological consequences in how metabolic control is distributed throughout the pathway and, possibly, how branch points in the pathway are coordinated. We therefore sought to investigate this hypothetical relationship further.

Numerous points along the conversion of glucose to lactate occur whereby the substrate can be diverted to another pathway and thus another end product. In fact, many have proposed that enhanced glucose metabolism observed in cancer cells is a consequence of a metabolic adaptation to increase the diversion of glycolytic intermediates to biosynthetic pathways[1,3,30,31]. To investigate the degree to which glycolytic flux can be diverted to a product other than lactate, we compared the glycolytic enzyme intensity levels with the levels of branch point (BP) enzymes acting on the same substrate (Fig. 3d). We found that throughout glycolysis the expression levels of the BP enzymes were significantly lower than the enzymes corresponding to the main pathway. Interesting however, was that at certain branch points, such as one emanating from 3-phosphoglycerate, the expression of the enzyme that catalyzes the committed step to the branching anabolic pathway was comparable and in some cases commensurate with the expression of the corresponding enzyme in glycolysis. Notably this occurs at the phosphoglyceromutase (PGAM) step in glycolysis in which 3PG can continue to be metabolized along glycolysis or it can be diverted to de novo serine synthesis by oxidation via phosphoglycerate dehydrogenase (PHGDH). Though on average the concentration of PHGDH was lower, in many cancer cell lines, the concentration of PHGDH was comparable or even higher than that of PGAM in some cases.

### Global Branch Point Analysis

**Fig 4:**
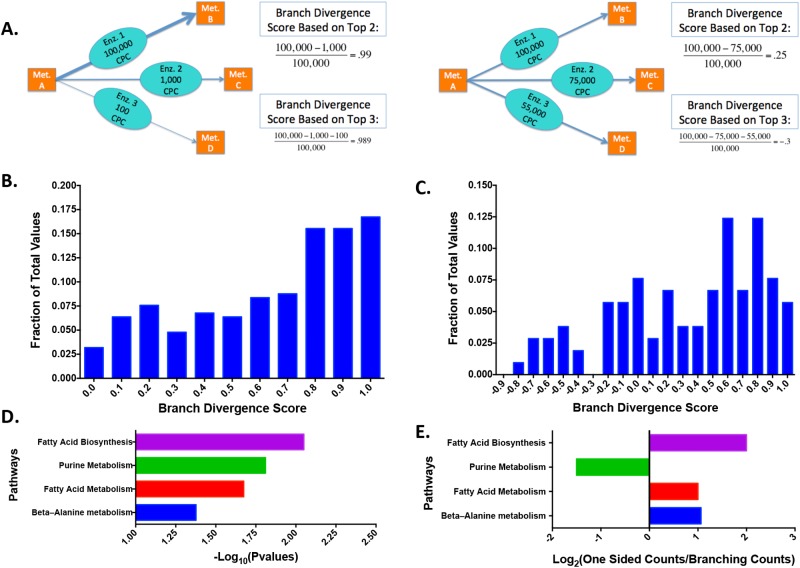
Branch point analysis across the human metabolic protein network. **(a)** Diagram clarifying method for computing various Branch Divergence scores in 2 different circumstances. Orange squares indicate reactant and product metabolites with blue ovals indicating the reacting enzymes. **(b)** Histogram of Branch Divergence Scores based on top 2 values. Each bin is lower end inclusive with a bin size of 0.1. **(c)** Histogram of Branch Divergence Scores based on top 3 values. Each bin is lower end inclusive with a bin size of 0.1. **(d)** Plot indicating the p-values for pathways that have significantly different counts in one-sided and equally distributed pathways (defined as a p-value < 0.05). **(e)** Ratio of one-sided counts to equally distributed counts for significant pathways.

Given the interesting findings we observed in glycolysis, we next sought to systematically assess protein concentration structure across the human metabolic network. In order to analyze the relative levels of BP enzymes outside of the core glycolytic pathway, we utilized the Recon 2 database to extract all points in the known metabolic pathways where a BP occurred. The BPs were filtered to include only those that contained proteins detected in the quantitative proteomics dataset. This filtering resulted in 251 BPs with at least two measured enzymes, and 105 with at least three. For each BP, we calculated two separate Branch Divergence scores (Fig. 4a, see Methods) based on either the top two or three most highly expressed proteins involved in the branch. The first score considered the fraction of protein concentration at the highest abundant protein involved in the branch compared to the second highest protein. The second considered the fraction of protein concentration at the highest abundant protein involved in the branch compared to the second and third highest protein. An inspection of these scores (Fig. 4c) revealed a non-Gaussian distribution with a peak near one for the first metric (Fig. 4b). For the score that considered the two highest expressed proteins, it was found that the distribution peaked at one indicating that the most common occurrence in cases BPs along the human metabolic network was when protein levels were concentrated along one predominant route in metabolism. However, it was also observed that many exceptions exist as the distribution exhibited a long tail with sufficient density near zero. An inspection of the histogram for the second metric (Fig. 4c) revealed a similar structure with the clear distinction showing that, in the tail, protein concentrations are distributed along multiple enzymes and thus through multiple routes. We next investigated which pathways tend to have proteins distributed evenly across branch points and which tended to have the expression occurring at a single route. It was found that for branch points with a predominant route, pathways involving fatty acid biosynthesis, fatty acid metabolism, and alanine metabolism were most commonly observed. This observation is evident in both the statistics (Fig. 4d) and the ratio of counts (Fig. 4e). For pathways with protein distributed evenly across branch points, the only statistical signal observed was in purine metabolism, with no other statistical signals for common pathways observed—suggesting that these hubs were scattered randomly across the whole metabolic network.

### Cofactor Analysis

Another valuable conclusion that can be drawn from this type of dataset would be how certain cofactors or crucial substrates are distributed across certain enzymatic reactions. We examined the distribution of enzymes that used certain cofactors, and considered an analysis of crucial cellular metabolic functions including the maintenance of redox potential involving both essential cofactors NADH and NADPH and the assimilation of nitrogen.

**Fig 5:**
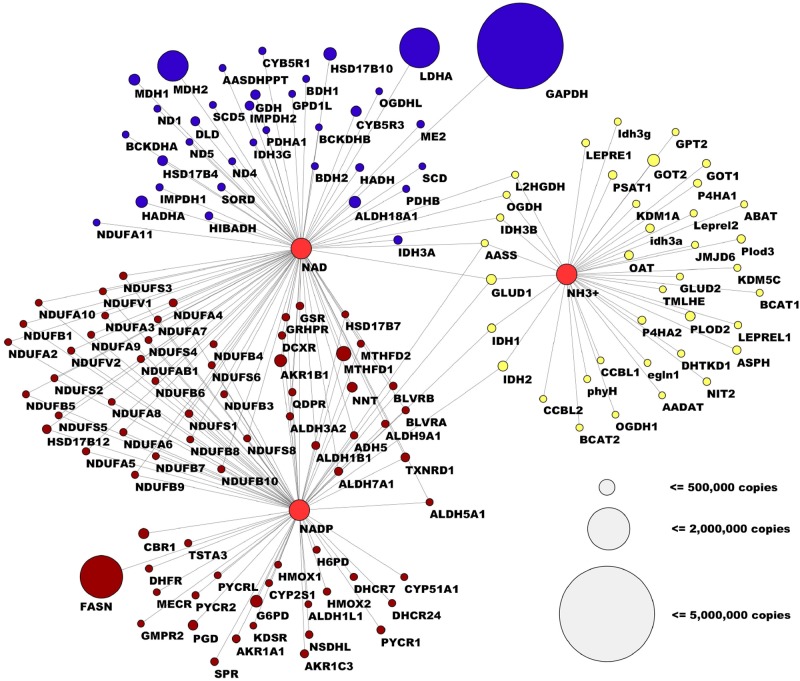
Cofactor and protein concentration analysis within the human metabolic protein network. Network diagram of glycolysis illustrating the abundance of aminotransferases and enzymes that utilize NAD(P)/NAD(P)H as a cofactor. The size of the nodes corresponds to the average abundance of the proteins.

We first attempted to analyze the relative usage of nitrogen, or which aminotransferases were most prevalent within a cell. Using the BRENDA database we isolated all aminotransferases that were detected within the dataset, and found that among the most abundant enzymes were GOT2, GLUD1, and IDH2 (Fig. 5). Together this finding identifies the key nodes in nitrogen assimilation. We next considered the utilization of cofactors involved in oxidation and reduction, NADP^+^/NADPH, and NAD^+^/NADH. We first considered NAD^+^/NADH and found, consistent with our observation that glycolytic enzymes constitute the most abundant enzymes in cells, that GAPDH and LDH comprised most of the protein concentration used for NADH-mediated redox coupled reactions in cells. Of lower abundance were those enzymes involved in the TCA cycle and biosynthetic and maintenance reactions in secondary metabolism. For NADPH, consistent with recent findings that have identified the oxidation of folates as a major source of cellular NADPH, the enzyme MTHFD is one of the most abundant enzymes that synthesize NADPH[32]. A major consumer of NADPH was fatty acid synthase (FASN), which is in line with the need for *de novo* lipid synthesis used in plasma membrane formation in rapidly proliferation cells. Both major consumers (LDHA, FASN) and producers (GAPDH, MTHFD and MDH2) of NAD(P)H are subject of major research efforts as drug targets against cancer[11,33–36].

### Correlations with kinetic parameters

**Fig 6:**
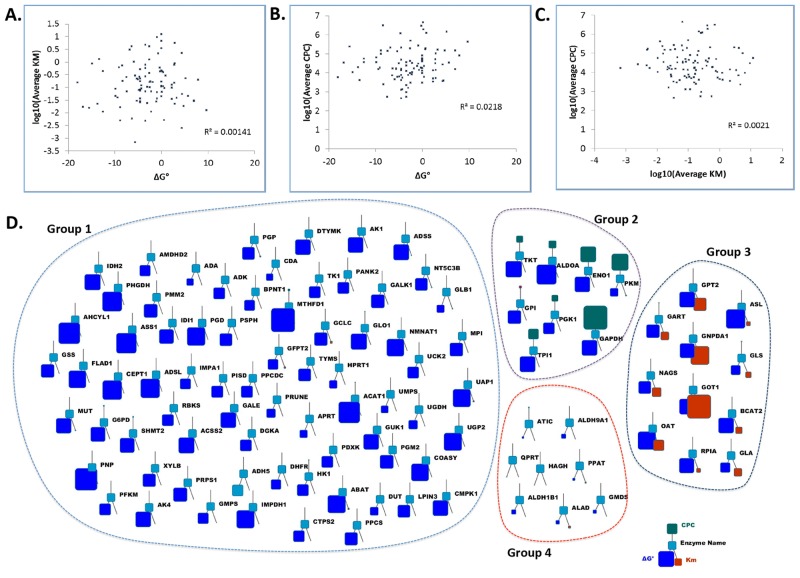
Analysis of enzyme concentrations in relation to kinetic and thermodynamic properties. **(a)** Scatter plot of the log of the average K_M_ and ΔG° value with each dot representing a different metabolic protein. **(b)** Scatter plot of the log of the average cell protein copy (CPC) and ΔG° values with each dot representing a different metabolic protein. **(c)** Scatter plot of the log of both the average K_M_ and average CPC value with each dot representing a different metabolic protein. **(d)** Connectivity analysis of CPC, ΔG° and K_M_ values for the various enzymes.

Ultimately, we note that that the reaction rate or flux through a point in metabolism involves not only the enzyme concentration, but kinetic parameters and the thermodynamics of the chemical compounds involved in the reaction as well. These parameters include the Michaelis constant (K_M_) and standard Gibbs Free Energies (ΔG°) of the reactions (S2 Table). We therefore investigated the relationship between these three fundamental parameters. Surprisingly no correlation between average protein level and ΔG° (Fig. 6a), K_M_ values and ΔG° (Fig. 6b), and average protein level and K_M_ values (Fig. 6c) were observed. To gain additional insight into the relationship between these three variables, we also visualized these three variables together for each enzyme. Using this approach we distinguished 4 groups (Fig. 6d). The bulk of the enzymes visualized in this manner had moderate ΔG°, K_M_ values and protein copy numbers (group 1) and were therefore responsible for the overall lack of correlation between these three variables. This finding is in contrast to a previously held assumption that larger protein concentrations are required for reactions close to equilibrium[37]. Furthermore, this analysis also provides strong evidence that each of these fundamental variables for a cellular metabolic reaction is uncoupled allowing for independent tuning of these three parameters for the evolution of the human metabolic network. Also, this lack of correlation suggests that overall, protein expression is emblematic of reaction rate since the K_M_ and ΔG° for each reaction involving a given protein concentration appears uncorrelated. There were however a few exceptions to this general rule. First, the proteins in group 2 (Fig. 6d) corresponded largely to the previously mentioned glycolytic proteins with no apparent large K_M_ or very low ΔG° values. This corresponds with the notion that these proteins need to be highly expressed to ensure a high glycolytic flux that may result in the buildup of glycolytic intermediates and subsequent enhanced flux into the various biosynthetic branches. The other two groups (group 3 and 4) contained enzymes with either very large K_M_ values or very low ΔG°. The extreme K_M_ values indicate that these enzymes need a substantial buildup of substrates in order to result in an appreciable forward flux while reactions with very low ΔG° are highly irreversible reactions. Indeed, three enzymes with large K_M_ values (GNPDA, GPT2 and GART) directly drain glycolytic intermediates into biosynthetic pathways while three enzymes with very low ΔG° (ATIC, PPAT and QPRT) utilize phosphoribosyl diphosphate (PRRP) derived from the pentose phosphate pathway for NAD(P)H and nucleotide synthesis. Also several enzymes in group 3 (GLS, NAGS, OAT, GOT1 and ASL) are involved in arginine, aspartate, glutamine metabolism, for which the metabolites have some of the highest intracellular concentrations and/or fluxes.

## Discussion

It is important to note that these analyses were performed on data collected from a panel of cancer cell lines rather than human tissue and thus the results of this analysis is prefaced with a series of caveats. While analyses on cell lines can often shed light on overall cellular understanding, there is great metabolic diversity across different tissue types that could affect the results of any global proteomic analysis. For instance, cardiomyocytes are thought to extract a majority of their energy from fatty acid oxidation and approximately 50% of their volume is occupied by the mitochondria [39]. Additionally, it has been shown that the media conditions used in tissue culture have substantial if not dominant effects on cell metabolism and influence enzyme activity[38–40]. Thus conditions specific to the cell line and microenvironment alter the distribution of proteins. Nevertheless, an analysis of protein abundance across the network provides insights into specific examples of how mammalian cancer cells distribute their enzyme concentrations

Overall, we considered an analysis of protein concentration of metabolic enzymes across the human cancer cell metabolic network in a diverse number of cells. This analysis reveals that a relatively simple calculation and assessment could yield multiple insights into the organization of the cancer metabolic network. Contrary to previously held notions, metabolic enzymes do not appear to be any more highly expressed than any other protein in cells. The major exception is glycolysis, which accounted for up to 10% of the entire cellular proteome. The remarkable allocation of protein concentration for a single metabolic pathway underscores its centrality in the utilization of the major macronutrient, glucose, which is used as an energy source. It also provides insights into the large fluxes that are observed in glycolysis and how these fluxes can be so fast. The finding also calls into question many control mechanisms that have been proposed to regulate glycolysis. For example, it is likely, that in many cases, post-translational modifications such as acetylation or phosphorylation may not have major regulatory roles since their stoichiometry is limited by the small number of kinases and acetyltransferases that are expressed to perform these reactions. Nevertheless most metabolic pathways have a far lower abundance of protein concentrations suggesting that most enzymes in metabolism could be under the regulation of post translational modifications.

In analyzing branching points in the metabolic network, we found that most commonly, the concentration of proteins tended to be distributed across a continuous route suggesting that flux that branches from a pathway is likely to be a minor component of the overall flux. This principle is perhaps underscored by the distribution of protein concentrations at branch points in glycolysis–which tended to be lower than the abundance of the corresponding enzymes in glycolysis.

Cofactors revealed many expected relationships and identified the key enzymes involved in the partitioning of these critical metabolites. Notably for NADH, we found no enzymes outside of glycolysis that substantially contributed to the metabolism of these cofactors. For NADPH, we identified major utilizations involving folate metabolism and lipid synthesis and oxidation. For aminotransferases we were able to determine the major nodes of nitrogen assimilation with little surprise.

Finally, we emphasize again that our analysis does not directly make conclusive statements about flux distributions in cells. Nevertheless the concentration of enzymes does place limits on the flux through that point in metabolism and may also further provide evolutionary insight into structure of the metabolic network by identifies the capacity for flux through each node. Hopefully in future work, these limits could serve as further constraints of models of flux distributions in metabolism and could allow for more accurate modeling of cellular metabolism.

## Supporting Information

S1 Table Proteome profiles of the NCI-60 cancer cell lines. Table contains cell protein copy number’s (CPC’s) derived from the label-free quantification (LFQ) output generated using the MaxQuant [41] software package and originally described by Moghaddas Gholami et al.[19].
(XLSX)

S2 Table CPC, K_M_ and ΔG° values. K_M_ values were retrieved from BRENDA and ΔG° from Henry et al.[23].
(XLSX)
